# Enactive explorations of children's sensory-motor play and therapeutic handling in physical therapy

**DOI:** 10.3389/fresc.2022.994804

**Published:** 2022-10-11

**Authors:** Ragnhild B. Håkstad, Gunn Kristin Øberg, Gay L. Girolami, Stacey C. Dusing

**Affiliations:** ^1^ Department of Health and Care Sciences, Faculty of Health Sciences, UiT The Arctic University of Norway, Tromsø, Norway; ^2^ Department of Physical Therapy, University of Illinois at Chicago, Chicago, IL, United States; ^3^ Motor Development Laboratory, Division of Biokinesiology and Physical Therapy, University of Southern California, Los Angeles, CA, United States

**Keywords:** play, enactive theory, pediatrics—children, motor learning, qualitative study, physical therapy

## Abstract

**Introduction:**

In pediatric physical therapy, there is an ongoing debate about the use of therapeutic handling and its potential effects on motor learning. In this study, we build on enactive theoretical perspectives to explore the role of therapeutic handling in connection to children's sensory-motor play, engagement, and performance during a single physical therapy session.

**Material and methods:**

This is a qualitative study based on video observations of therapy sessions and interviews with 15 physical therapists (PTs) each treating two different children aged 0–3. The authors utilized a framework of co-reviewing, discussing, and reflecting on the sessions. Themes were identified and used to describe the ways by which PTs’ therapeutic handling unfolds, with connections to theories on sensory-motor play and learning, along with enactive perspectives on embodiment, experience, mutual incorporation, and sense-of-agency.

**Results:**

The characteristics and purposes of therapeutic handling are presented in two main themes: (1) position and support, and (2) directing movement. We found that position and support promoted sensory-motor improvement when the PTs’ handling aligned with the child's play interests and engagements. As part of play, the children used new and additional support surfaces to self-initiate better posture and movement solutions and reach play goals. The PTs’ ways of directing movements varied. To awaken curiosity and induce a child's self-driven motor exploration the PT needs to be subtle, flexible, and precise in the directing of movement. This entails responsiveness to the child's signals and bodily know-how in the placing of hands and direction of pressure to enable the child to actively participate in and eventually self-drive movement.

**Discussion:**

Therapeutic handling that is mutually incorporated between PT and child can enrich the child's playing-to-learn-to-move process by providing novelty and facilitating the child's sense-of-agency in the self-initiated exploration and refinement of movement possibilities. In the PTs’ effort to merge therapeutic handling with children's play, the momentum of interaction can open new therapeutic windows of movement experience and learning opportunities.

## Introduction

Bodily interactions, i.e., interaction of any part of the body with a person, objects, or the environment, are essential to us as humans and a natural part of how children learn to move. Across cultural settings, children interact with their environments, including caregivers, to understand and develop their own movement capacities (1). As part of these interactions, caregivers find ways to facilitate a child's motor skills and learning by providing support, guiding the direction of movement, and moving along with the child as he or she discovers and problem-solves new motor tasks. Daily life examples of such bodily interactions are sitting with support on a parent's lap or a gentle push or a hand for support to climb steps or ladders. In pediatric physical therapy, bodily interaction in terms of therapeutic handling is a dimension of therapeutic touch and a hallmark of clinical practice. However, the ongoing debate about therapeutic handling as part of the therapeutic work process has led to a dichotomizing view of hands-on approaches labeled as therapist-led and passive, as compared to hands-off approaches labeled as child-led and active (2, 3).

Historically, hands-on approaches with emphasis on therapist-led passive movement and forced positioning were developed on the basis of now outdated theoretical perspectives on disability, development, and learning (4–8). Hierarchical and neuro-maturational developmental theories with their understanding of reflexes vs. higher-level organization of movement, along with later motor programming theories with emphasis on movement synergies (5), led to the idea that children can and should learn to correct their motor performance according to specific or “correct” movement patterns. A paradigmatic change came with the introduction of dynamic system theories, in which motor development is viewed as the result of multicausal and self-organizing processes within the individual-task-environment system (5, 6, 9). In accordance with this, clinical practice is oriented toward problem-solving of tasks and expanded physical therapy beyond the clinic into the child's everyday environment. More recently, knowledge about child development and learning has evolved and neuro-scientific evidence points to the importance of attention, motivation, and self-initiation of movement (4, 10). This new knowledge indicates that new motor skills are learned when we engage children in motivating, play-oriented, and self-initiated movement activities (3, 4, 6, 11, 12).

The emphasis on self-initiation of movement is also the main argument for why hands-on treatment approaches are considered detrimental to motor learning (2). We question the foundation of the view that motivating, play-oriented, and self-initiated movement activities can only occur during hands-off therapeutic activities. We want to explore if these activities can also include hands-on approaches (4). Moreover, building on the view that therapeutic handling can be part of the child's learning through a bodily dialog between the physical therapist (PT) and child (1), we question if the child's playful learning opportunities can even be diminished if pediatric PTs abandon the use of hands-on approaches, thus reducing the children's natural movement learning as an interaction with other moving bodies. We approached this qualitative study as an in-depth exploration of the role of therapeutic handling in connection to children's sensory-motor play and performance during physical therapy. We explore the ways by which PTs’ therapeutic handling unfolds in various therapeutic situations and analyze the interactions between PTs and children, the children's play behavior, and sensory-motor performance.

### Theoretical perspectives

To further elaborate on the theoretical fundaments of our study we will present the concept of enactive therapeutic sensory-motor play, its theoretical underpinnings, and key terms applied in our analysis.

#### Enactive therapeutic sensory-motor play

In pediatric physical therapy, we treat children who have sensory-motor impairments, which affect their ability to play and learn. The core of pediatric physical therapy is to ameliorate these impairments and help children move, play, learn and participate as best they can in their enactment of their world. The concept of enactive therapeutic sensory-motor play is established by the first and second authors and seeks to unpack how PTs can enter, without disrupting, the children's play world and find ways to improve the child's playful learning as part of their therapeutic approach (13). To achieve this, the PT must find ways to incorporate therapeutic measures targeted to the child's specific motor impairments and combine these in engaging, interactive sensory-motor play activities. Fundamental to this successful merging of play and therapy is the development of shared intentions between PT and child. The PT must attend to the child's signs of intention, attention, and motivation so that therapeutic actions such as handling, choices of toys, and changes to the task or environment become “part of the play,” not a disturbance to it. This entails the incorporation of bodily know-how into the PT's clinical reasoning (1, 14), i.e., being sensitive to the child's bodily signals and accepting the child as an initiator and active agent of movement. As part of this concept, therapeutic handling can be a means by which children discover and explore new sensory-motor movement and play possibilities.

#### Theoretical underpinnings: sensory-motor play and learning

The abundance of neurons in a young child's neural system can be viewed as a jungle of connections that needs to be explored (15). Play can be viewed as a way for the child to explore and try out this jungle of possibilities (12, 16). During playful exploration, learning gradually takes place in terms of neural selection and consolidation of the connections most useful for the child (17). While such learning takes place in other settings as well, play is inarguably an important source and provider of the ingredients we know are crucial for learning, e.g., exploration, variation, repetition, and motivation. Recent findings also indicate that epi-genetic alterations take place during social and explorative play, opening up “the gateways of learning” (12). For children with impairments, there might be a smaller “jungle” for them to explore, i.e., their ability to perceive and process sensory experiences and their repertoire of motor actions, as well as their innate drive and ability to learn from these sensory-motor experiences, may be limited. However, as part of motor learning and development, it is important for children to play their way through their test ground of neural connectivity possibilities.

In concordance with dynamic systems theory, Adolph and Robinson (18) proposed an ecological *learning-to-move-and-moving-to-learn* process based on children's exploration and problem-solving of movement possibilities in their environments, through which children learn about their own body and what it is capable of. Sheets-Johnstone (19) takes a phenomenological perspective on these same behaviors. She emphasizes our primary and innate drive for pleasurable, playful exploration of our sensory-motor possibilities, and how this playful exploration serves learning about ourselves as bodily beings. Combining these perspectives, children's learning-to-move-and-moving-to-learn is indeed also a *playing-to-learn-to-move* process, and fundamental to the way children enact and learn about their world through play. For the PT, these perspectives on children's sensory-motor play and learning entail that it is an important therapeutic task to find ways to expand the children's movement experiences and actions and help them discover and self-direct their play and learning processes.

#### Theoretical underpinnings: enactivism

In this section, we will present the enactive perspectives that underpin the concept. Enactive theory is a merging of theoretical and philosophical perspectives predominantly from dynamic systems theory, neuroscience, and phenomenology of the body. It offers new perspectives on how we enact our world, i.e., how our bodily experiences and actions form our cognition and understanding of ourselves and our physical, social and societal surroundings (20). For the purpose of this paper, we will focus on selected terms in enactivism that serve to explain how bodily interactions and therapeutic handling connect to children's motor development and learning.

*Embodiment* and *experience* are two core terms in enactivism (20). Embodiment as understood in enactivism highlights the way we act and learn, through a continuous brain–body–environment system, in which movement initiatives and perceptions of the body and environment co-exist and co-inform our development. The nervous system is a continuous sub-system within a brain–body entity that perceives and acts upon its environment both simultaneously and holistically. Experience then molds us as individuals and is the foundational source of learning within this brain–body–environment system (1, 20). It is a transformational force through which we develop and refine new skills in interaction with the world. When we connect to motor development and learning theory (4, 17, 21), experiences of various movement initiatives, actions, and perceptions of the body and environment serve to expand the repertoire of ways children act and interact with their world. Furthermore, as experiences are repeated and learning and transformation proceeds, the most efficient or preferred ways to act and interact can be advanced and refined.

**Figure 1:**
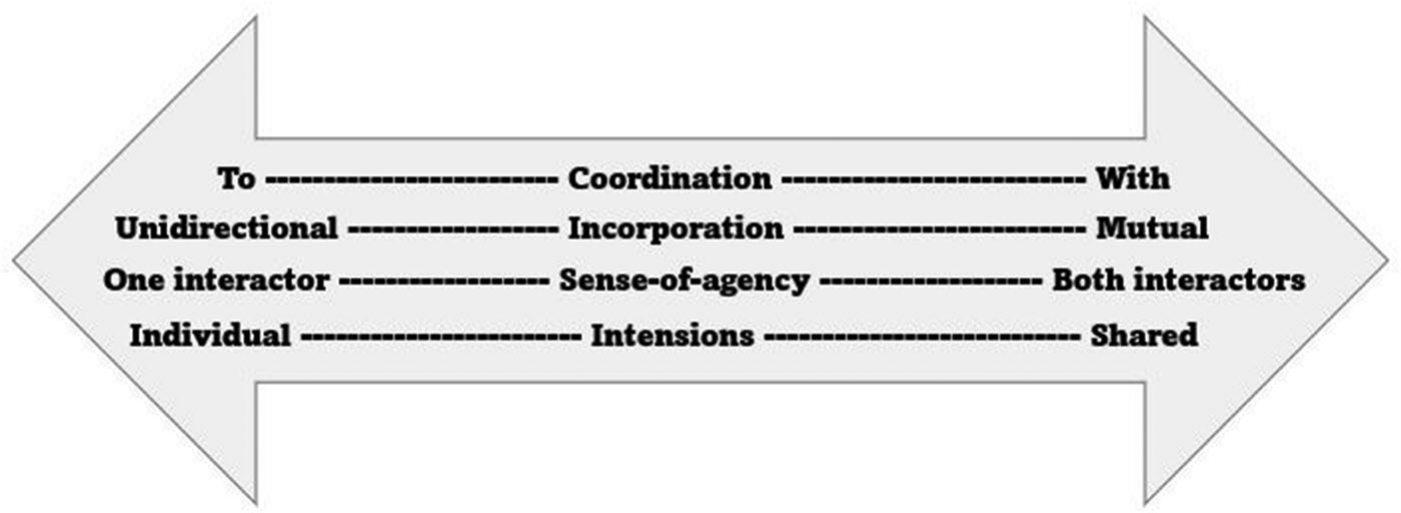
The continuums of the enactive terms coordination, incorporation, sense-of-agency, and intention.

The phenomenological term *mutual incorporation* (22) is particularly relevant for the understanding of therapeutic handling. This term highlights the dyadic interplay that arises when we engage in bodily interactions. Starting out with individual intentions, each interacting body oscillates between being the active or the receptive part during any interaction. It is predominantly *unidirectional incorporation* in which each interacting body will be the active agent or receptive to the actions of the other (22). Whereas when the interactors share intentions, they can engage in a common intercorporality in which both parties act and receive the other's action simultaneously and in mutuality. A similar distinction can be found in the enactive understanding of our bodily *coordination to* as opposed to *with* another moving body. Bodily coordination with another body requires these interactive oscillations to be diminished so that one of the actors is unidirectionally leading and the other is following the lead of action. Whereas coordination with another body entails synchrony of oscillations in which both interacting bodies get to act and react upon the other's moving body, in concurrence with their own intentions (22). It is important to note that these are theoretical distinctions that in real-life interactions will usually fluctuate between unidirectional and mutual incorporation, or between coordination to and with, and with the agents taking turns being the active or the receptive part. Finally, we also want to highlight the term *sense-of-agency* (1, 23). This phenomenological term pinpoints the bodily perception of being the one who performs a movement or action, as opposed to being moved by something or somebody else (or being unaware of the moving agent). Connecting to motor learning theory (4, 17), sense-of-agency resonates with the self-initiation of movement that is fundamental for learning. The term underscores that interactions in which the child is the receptive part are less likely to provide the child with a sense-of-agency, and thus less likely to provide the child with transformative learning experiences. Figure 1 shows the different terms and how they can be understood as similar continuums along a scale.

Returning to therapeutic handling, these perspectives can lend some support to the labeling of hands-on approaches as therapist-led and passive, i.e., in interactions in which the PT is unidirectionally coordinating to rather than with the child, which in turn can diminish the child's sense-of-agency. But more notably, they support the idea that therapeutic handling is multi-faceted and dynamic in nature. Hands-on approaches can be part of interactions with mutual incorporation and coordination with each other, including a sense-of-agency for both parties. Furthermore, there can be interactions including therapeutic handling in which the child is the active part while the PT focuses on being receptive and responsive to the child's actions. Or the other way around, therapeutic handling in which the PT is the most active and the child is the most receptive part can still provide the child with bodily experiences that he or she can turn into future action.

## Material and methods

This is a qualitative study based on video observations of therapy sessions and supplementary interview data with PTs treating children aged 0–3. Our methodological standpoint is hermeneutic with phenomenological traits and aligns with the enactivist theoretical stance. We aim to clarify and acknowledge our preconceptions, emphasize the experiential and interpretive processes discussed by the research group, and develop common ground and new levels of understanding by the research team (24, 25).

### Study design

The study was designed to recruit pediatric PTs in Norway and United States who indicated they used play as part of their therapeutic interventions. Each PT was asked to recruit two families willing to participate in the study. Data collection consisted of video observations of therapy sessions with the two children, followed by an interview with the PT which includes reviewing selected clips from the video recordings of the therapy sessions.

### Study context

The choice of study sites in two different countries, including two different states in the United States, was made to obtain richness in the data material. We wanted to include PTs with different cultural and educational backgrounds and with a variety of clinical practice settings. However, the researchers’ existing collaborative networks and the preference for Norwegian or English languages were also decisive in the choice of study sites and make the study mainly representative of western clinical practice settings. This is confirmed by our impressions of the data material. Despite differences in the organization of healthcare services between Norway and United States, provision of services, therapeutic styles, and knowledge foundations in this study had individual variations but these appeared to be similar across sites. The most important difference across sites is that all sessions in the United States were in the family's home, while all sessions in Norway were either at the PT's treatment site or in the daycare setting. In addition, the PTs in the United States should make use of what was already in the home and be discouraged from bringing their own equipment into the home.

### Recruitment and participants

In Norway, recruitment took place *via* the Norwegian Physiotherapist Association (NFF)'s pediatric section. The section administration sent emails to their members, and we were allowed to post information about the study on their social media platforms. PTs that wanted to participate in the study contacted the researchers at their own initiative. In the United States, research team members recruited pediatric PTs from the local community. They sent emails to their collaborative networks of pediatric PTs in their area, inviting them to respond at their own initiative if they wished to participate in the study.

Seven PTs in Norway and eight PTs in the United States were included in the study (*n* = 15). Thirteen PTs recruited two children aged 0–3 who received physical therapy on a regular basis. Of the remaining two PTs, one recruited only one child and the other PT had three participating children: one individual session and one group session with two children together. A total of 15 PTs and 30 children participated in the study with a total of 29 sessions. The content of sessions varied, in accordance with the children's functional levels and interests as well as the PTs’ therapeutic style. Out of the 29 sessions, 16 sessions contained any type of therapeutic handling in connection to play and were included in the analysis. The remaining 13 sessions, which did not contain any situations of therapeutic handling in connection to play, were excluded. This absence of handling was either due to the PT's choice of approach, the child's functional level making handling irrelevant, or sessions that were discontinued because of the child's health status that day.

The clinical experience of the participating PTs ranged from 3 to 26 years, and they all had pediatric training in addition to their physical therapy education. All PTs stated they primarily used an eclectic therapeutic approach. In addition, two PTs had Vojta training/inspiration, one was trained in Neuro-Developmental Treatment (NDT), one emphasized the use of biomechanical principles, and one used a psychodrama approach.

The participating children were evenly spread across the age range 0–3 with diagnoses ranging from mild motor delays to severe developmental disorders. We have categorized their functional levels to

(1) Floor mobility not developed—6 children, 4 included in the analysis.
(2) Some floor mobility and or independent sitting—4 children, 2 included in the analysis.
(3) Standing/cruising/stepping—12 children, 9 included in the analysis.
(4) Independent walkers—8 children, 2 included in the analysis.

The low inclusion of independent walkers may indicate this group was less likely to receive therapeutic handling. See Tables 1 and 2 for more details about the participants and the inclusion and exclusion of sessions.

**Table 1:** Overview of the participating children's functional level and age, and the inclusion/exclusion decisions.

	Functional level (Cat. 1–4)	Age	Inclusion/exclusion
C2	1: Floor mobility not developed. Initial grasping in supine	4 months	Handling in both categories
C6	1: Floor mobility not developed. Handles toys with both hands	8 months	Handling in both categories
C15	1: Floor mobility not developed. Can handle toys, best with her right hand	2 years	Handling in both categories
C17	1: Floor mobility not developed. Handles toys with both hands	7 months (CA 5 months)	Handling in both categories
C11	1: floor mobility not developed. Initial grasping skills	5 months (CA 3, 5 months)	Excluded—not sufficient material
C12	2: Crawls, sitting with support	10 months?	Handling in both categories
C28	2: Can sit for short periods. Rolls from prone to supine	8 months (CA 5 months)	Handling in both categories
C26	2: Can sit independently. Crawling. Some standing with support (but crouched/leaning forward)	14 months	Excluded—not sufficient material
C29	2: Pivots in prone	5 months	Excluded—little handling
C5	3: Stands with support, creeps/crawls, sits independently	1 year 6 months	Handling in both categories
C9	3: Cruises/walks with support	18 months	Handling in both categories
C16	3: Sits and stands with support, crawls, handles toys with both hands (best with her right)	11 months	Handling in both categories
C18 + C19	3 + 3: Both sit and stand with support and handles toys. One with crawling skills, one crawls with support. Both use walkers	2 years + 2 years	Handling in both categories
C20	3: Child crawls, stands with support, handles toys	2 years	Handling in both categories
C22	3: Crawling and cruising. Sits independently. Handles toys with both hands	12 months	Handling in both categories
C24	3: Sits independently, cruises	1 year 2 months	Handling in both categories
C30	3: Creeping, stands with support, cruising Sits independently	11 months	Handling in both categories
C1	3: Cruising, walks with support	1 year 7 months	Excluded—no handling
C3	3: Cruises/walks with support	2 years 1 months	Excluded—little handling
C4	3: Child cruises/walks with support	11 months	Excluded—no handling
C13	3: Cruising	10 months	Excluded—little handling
C21	4: Independent walker	2 years	Handling in both categories
C23	4: Independent walker	1 year 6 months	Handling in both categories
C7	4: Independent walker	3 years	Excluded–no handling
C8	4: Independent walker	2 years	Excluded—no handling
C10	4: Independent walker	2 years	Excluded—no handling
C14	4: Independent walker	2 years	Excluded—no handling
C25	4: Independent walker	2 years	Excluded—little handling
C27	4: Independent walker	2 years 6 months	Excluded—little handling

CA, Corrected Age.

**Table 2:** Overview of participating physical therapists and the inclusion/exclusion decisions.

	Therapeutic approach	Inclusion/exclusion
P1-C1	Eclectic/Vojta inspired	Excluded—no handling
P1-C2	Eclectic/Vojta inspired	Handling in both categories
P2-C3	Eclectic	Excluded—little handling
P2-C4	Eclectic	Excluded—no handling
P3-C5	Eclectic/Vojta inspired	Handling in both categories
P3-C6	Eclectic/Vojta inspired	Handling in both categories
P4-C7	Psychodrama approach	Excluded—no handling
P4-C8	Psychodrama approach	Excluded—no handling
P5-C9	Eclectic	Handling in both categories
P6-C10	Eclectic	Excluded—no handling
P6-C11	Eclectic	Excluded—not sufficient material
P7-C12	Eclectic	Handling in both categories
P7-C13	Eclectic	Excluded—little handling
P8-C14	Eclectic	Excluded—no handling
P8-C15	Eclectic	Handling in both categories
P9-C16	Eclectic	Handling in both categories
P9-C17	Eclectic	Handling in both categories
P10-C18 + C19	Eclectic	Handling in both categories
P10-C20	Eclectic	Handling in both categories
P11-C21	Eclectic	Handling in both categories
P11-C22	Eclectic	Handling in both categories
P12-C23	Eclectic/NDT inspired	Handling in both categories
P12-C24	Eclectic/NDT inspired	Handling in both categories
P13-C25	Eclectic	Excluded—little handling
P13-C26	Eclectic	Excluded—not sufficient material
P14-C27	Eclectic	Excluded—little handling
P14-C28	Eclectic	Handling in both categories
P15-C29	Eclectic	Excluded—little handling
P15-C30	Eclectic	Handling in both categories

NDT, Neuro-Developmental Treatment.

### Data collection

The first author, the principal investigator (PI), conducted the data collection. During the initial visit, the researcher gave a brief verbal description of the study, obtained informed consent from the PT and the family, and encouraged the participants to proceed with sessions as usual. Video recordings were done with a small hand-held camera. The PI was positioned in the background and made every effort not to disturb the participants. If children made contact and wanted to interact with the PI, she gently confirmed with smiles and small-talk, but then withdrew from the interaction as soon as possible. After the session, there was a short debrief to invite the participating PT to highlight sections they would like to view during the follow-up interview.

The interviews were conducted within 24 h after the completion of both observations (in one case the observations were split over two visits and required two separate interviews), at a time and location convenient for the participating PT. The interview guide began with questions on the PT's background and general thoughts on the use of play in physical therapy. This was followed by a short conversation about each of the children observed during the PT sessions. After this, elicitation of a more detailed discussion of therapeutic events was initiated based on viewing selected clips from sessions (26). This selection was based on a mix of the PT's requested sections, and the researcher's identification of interesting play situations.

### Analysis

After data collection was completed, workshops with the researcher team and user representatives (parents and pediatric PTs) were arranged, to view sections of the video based on the initial research questions. During the review of videos, observations and discussion led to evolving research topics. In parallel to this, the first author transcribed data and performed an initial coding and sorting of the data material using NVivo software (RRID: SCR_014802). Initial written transcripts of sessions gave a summary of each session along with reflections related to the merging of play and therapy. Interviews were transcribed verbatim, serving only as supplementary data in this current study and will be further explored in future studies.

The topic of therapeutic handling emerged from both initial analysis steps. In the coding of the written transcripts, i.e., a labeling process inspired by Malterud (27), therapeutic handling emerged as one of the main topics in the material. During workshops, the identification of the range and differences of therapeutic handling events inspired us to explore these variants more thoroughly. The first author reviewed the data material for relevant sessions and selected video clips for more focused reviewing in subsequent workshops with the research team. Based on these discussions the first author started to outline the results section and presented in this paper, and the selection of examples that best depict our overall findings was part of this process. Inspired by elements in the PRISMA method (24) for investigating interaction and intersubjectivity, we also analyzed four of the examples in this paper (Adrian, Beatrice, Charlotte, and Felix) to develop a deeper understanding of both the PTs’ and the children's interactional experiences. The ongoing analysis with connections to theory and discussion of findings have been further evolved during research team discussions and drafting of the manuscript.

### Methodological considerations

In our evaluation of the research process, we used the EPICURE agenda (28). In this acronym, E is about engagement. The authors are all pediatric PTs with clinical experience from primary and secondary care settings, either before or in parallel to our current academic positions. We are all engaged in the understanding, development, and critique of clinical practice in the pediatric field, with a special interest in the connections between mind-body-world and the co-emergence and co-dependence of motor and cognitive development. The first and second authors are also the creators of the enactive therapeutic sensory-motor play concept and their engagement in the application of enactivism has been decisive to the deductive theoretical stance at the onset of the study.

*P* in the acronym refers to the processing of the data material. The first author collected all data and conducted all transcriptions, including translations from Norwegian to English when necessary. She also selected video clips both for the interviews and for reviewing during workshops and conducted the coding of the material in NVivo. Transparency of these processes is provided by the presentation of the steps of analysis, the nuanced presentation of findings, and the overview of included sessions provided as supplementary material.

I in the acronym stands for interpretation. While the preliminary analysis and coding were conducted by the first author, rich discussions of the data material in workshops with user representatives and within the author's group ensured the voicing of varied interpretations and perspectives when viewing the material. Regular meetings and revision of drafts as a group effort have also ensured the development and agreements among the authors on how to interpret the data.

C for critique relates both to the research process and products, and the value of its outcomes. As a critique, we acknowledge that the study is based on a preconception that therapeutic handling is a valuable tool in physical therapy that can facilitate play and learning. We acknowledge this is the lens through which we viewed and analyzed the data material. Nonetheless, we also recognize therapeutic handling is not always positive, and our goal has been to explore and describe the details and nuances in interaction that demonstrate the differences between the two. The study's value lies in the ability to shed new light on the understanding of therapeutic handling by the use of enactive theory, through which we also expand and develop the theoretical underpinnings of pediatric physical therapy.

U for usefulness and R for relevance are closely related phenomena. We perceive our study as highly useful and relevant both for pediatric PTs in clinical practice and for the research community. Our aim is to initiate reflections and debates among clinicians and researchers. To ask them to reflect on the nature and conduct of therapeutic handling of children, with the hope of expanding their knowledge and contributing to their understanding of bodily interactions in pediatric physical therapy.

Finally, E is for ethics. There are many aspects to consider. The study was approved by the institutional review boards (IRBs) at Virginia Commonwealth University (VCU), the University of Illinois at Chicago (UIC), and the Norwegian Centre for Research Data (NSD) at UiT The Arctic University of Norway (UiT). Data are confidentially stored in accordance with institutional policies. Another ethical concern was asking participating PTs to recruit families for the study. We emphasized that they should inform families about this as an opportunity, but not in any way persuade or coerce them to participate. Those willing to participate received information letters and gave written consent on IRB-approved consent forms. Only parents consented on behalf of the children because of their young age. To ensure the ethics of including these children in the study, the researcher was attentive to the children's reactions to her presence during data collection and prepared to discontinue data collection if a child objected to her presence (29). No such events occurred, and data collection proceeded as planned. We emphasize the anonymous presentation of participants. In our presentation of results, we have given the children fictive names in alphabetical order and presented all PTs as female. We have also removed identifiable information to the best of our ability and expect only the participants themselves to be able to recognize individual examples as their own. In our presentation of results, there are examples and interpretations of what we perceive as interactional mismatches or breakdowns. We want to underscore that these are not representative of the individual PT's overall approach, but they are important events that highlight the discrepancies between more and less successful bodily interactions in the observed sessions.

## Results

Two main characteristics and purposes of therapeutic handling during play and intervention were identified: (1) Position and support, and (2) Directing movement. There are many ways by which the PTs proceeded with these handling purposes as part of play and intervention. In our presentation of findings, we will focus on these variations, i.e., the nuances in the interactions between the PT and child when it comes to the PTs’ handling and the child's responses to them, and how they relate to the child's sensory-motor performance. We will further show how these variations relate not only to the PT's handling approach, but also to the child's play initiatives and engagement in association with the PT's goals and clinical reasoning during the sessions.

### Position and support

The PTs used their hands to place and support the children in supine, prone, 4-point kneeling, sitting, and/or standing positions to play. One of the PTs also included prone and seated positions on a therapy ball. Position and support of the child also take place as a preparation for or during transitions, e.g., from side lying to sitting, in which the PT positions an elbow to enable weight-bearing and supports the trunk as the child moves to sit. These position and support actions can be due to the child's own play initiatives, or they can be part of activities the PT introduces to improve motor function and develop new movement skills.

In the videos, there are situations in which the PT's position and support hinder the child's active movement exploration and lead to objections and disengagement from play. This occurs when the PT places a child in a position with postural demands which are too high for his ability, and with the aim of working in a fixed position over time rather than exploring movement variations and play. On these occasions, an unfavorable struggle arises between the PT and child, and the child's attention switches away from play and over to the intrusive handling and difficult postural or movement demands that play now entails. Here is a situation that exemplifies such a struggle, leading to a breakdown of play and non-productive motor activity. Adrian, an 8-month-old boy (P3-C6) with motor delay who has not yet developed elbow support in prone is challenged by the PT in a modified 4-point position with his knees and hips flexed more than 90° and under his torso and his elbows on a soft wedge and with a mirror in front.

*Adrian looks and babbles into the mirror, leaning heavily onto the wedge with the PT's hands supporting his shoulders. He then reaches his left hand to touch the mirror. The PT acknowledges his action, saying “Did you feel him there?”, but then quickly takes hold of his arm and brings it back to elbow support. Adrian complains and collapses on the wedge, but then sees himself again in mirror and smiles and reaches for it a second time. The PT helps him back up to the initial position and Adrian reaches a third time for the mirror but the PT who restrains his arm to maintain elbow support. After a short struggle including fussing and increased non-productive motor activity the PT aborts the activity, saying “Oh boy, oh boy, I’ll put you down then”*.

During the interview, the PT recognizes Adrian's repeated reaching toward the mirror and says that they are easy to see on tape, but that she did not really notice them during the session: “*Because I was too busy with my own doing, you know*.”

Most of the time, such disconnects can be resolved and play opportunities can arise as they work their way through the struggle and reflect on why a situation may be failing. To exemplify this, we present a situation with 2-year-old Beatrice (P8-C15) with severe motor and cognitive delay, placed in a kneeling position with her chest heavily leaning onto the right hand and arm of the PT:

*Beatrice is playing with her favorite light-move-and-sound-toy, reaching with her right hand to push the buttons that set the toy off. She is fully engaged in the activity and her face shows this engagement with a smile and intense visual awareness. The PT wants Beatrice to bear weight on her left hand, which is up in the air. Play is disrupted as the PT lifts Beatrice away from the toy and tries to open her fist. “Can we get this hand down, these fingers out?” the PT says. Beatrice grunts and moans. “Am I making you mad?” the PT asks, then continues, “I am, can I have that hand?”. Beatrice grunts again, but the PT persists and is eventually able to open the hand and bring it to the floor. “Got you!” she says. With the left hand now supporting some of her weight, Beatrice immediately stops grunting and re-engages with the toy using her right hand. The PT encourages her play: “Go get it, get it! Good! Good job!”. After a short while her left arm flexes off the surface again, but the PT takes hold of it and is able to bring it back down with less of a struggle. Beatrice again refocuses on the toy, moving her right hand to different places on it and following her own actions with her gaze*.

In the interview, the PT explains that she is giving maximal assistance and support to enable play and motor performance in this position: “*If stabilized enough she will lift her right hand pretty well*.” She admits Beatrice would probably manage weight-bearing on her right arm better, but it was not a priority because play would then not be an option: “*She wouldn’t have lifted with the left arm*.*”*

Finally, the videos show many examples in which the position of the child and the support provided increased the child's play engagement and promoted the child's discovery of new or improved motor actions. In these examples, the PTs aim to provide the children with a better foundation for active, self-initiated movements and the exploration of new ways to find and use support surfaces. They can also introduce additional surfaces of support that the children can use to discover new or more efficient posture and movement solutions and reach play goals. The PTs emphasize the children's active exploration and provide bodily support as an integral element of play so that it expands the children's movement and action possibilities. During this work, the PTs synchronize their movements with the children's moving bodies and make frequent adjustments when it comes to where, when, and how to provide support. Adequate timing and the amount of support help the children complete play tasks and increase their motivation and engagement during play. As the children move, the PTs’ continued synchronized support, or readiness to support, provides a “safety net”; the children become better able to tackle new movement challenges when there is something to lean on or safely fall upon. Just as important in this synchronization is knowing when to withdraw support and allow for the children to reclaim the responsibility of independent postural control and movement.

Our example is 11-month-old Charlotte (P9-C16) with motor delay and greater involvement on the left side of her body. She can crawl but cannot sit or stand without support. The situation begins with the PT kneeling on the floor with Charlotte on her lap while she puts on her tights:

*The PT involves Charlotte, first by talking to her about placing her feet inside the tights and next by making her transition to standing to pull the tights up while verbalizing, “On and up with those tights, now you can stand for a bit, right?”. The PT coordinates her movements with that of Charlotte, rising herself to upright kneeling to support Charlotte as she transitions to stand. Charlotte has a flexed trunk and is quite busy fiddling with a string of beads but is cooperating in moving up to stand allowing the PT to pull up her tights. Charlotte now stands with mild flexion in hips and knees and leaning back against the PT's thighs. As she drops the string of beads to the floor, she extends her trunk, and the PT can remove her hands off Charlotte's body so that her only support is that of the PT's body behind her*.

*The PT now picks up the beads with her left hand and dangles it above and to the left of Charlotte's head, with her right hand supporting on the front of Charlotte's trunk. Charlotte turns her head to the left to look for the beads and reaches with her left arm but has difficulty aiming at the moving target. “Do you want it?” says the PT and moves her right hand gently onto Charlotte's chest to give her increased support simultaneously holding the beads still. Charlotte grasps the string of beads and then leans more into the PT's lap, fiddles briefly with the beads before dropping them back on the floor. The PT gently strokes Charlotte's left arm and leg, while saying “You can stand and reach when you have some support from behind”, then turns to mom “… she was able to stretch quite well”. Mom responds, “Yes well, that*'s *how it is, she does need support to be able to do things”*.

*Now the PT repositions Charlotte's feet before presenting the beads again on the left side. This time, Charlotte is seated on the PT's lap. Charlotte rotates her trunk, reaches, and grasps the string of beads, and fiddles with while in the seated position. Now the PT explores different adjustments of Charlotte's sitting height and pelvis tilt and removes her hands as soon as Charlotte can maintain an upright posture of her back. Charlotte again drops the bead string, and the PT retrieves them placing them on a low table on Charlotte's the left side. Charlotte rotates her trunk, shifts weight onto her left foot, and reaches and grasps the string, and the PT says “You can do it!”. After sitting back and fiddling with the string for a short while, the PT allows Charlotte to slide gently off her lap into floor sitting*.

During the interview, the PT explained that this situation was spontaneous, arising, and evolving by watching the child and her interest in the beads and by their moving together between sitting and standing positions. It was important for the PT to use her own body to support Charlotte, because: “*then I feel how much and how important (support) is needed. I worked together with her in rising to stand, and I was able to follow her all the way up to her reaching. It just came naturally*.”

Summing up, the PTs’ ways of positioning and providing support promotes sensory-motor improvements when the PT's actions align with the child's play interests and engagements. Failure to notice a child's play intentions can easily lead to interactional breakdowns and a child that resigns from play and or object to the PT's handling. This attentiveness to the child's play engagement corresponds with the distinction and fluctuations between coordination to and with, or unidirectional and mutual incorporation. The PT who was “*too busy with my doing”* in her handling of Adrian can be interpreted as being at one end of the scale, the mutual bodily interaction between Charlotte and her PT which “*just came naturally”* at the other, and the interactions during the PT's handling of Beatrice as fluctuating between the two.

### Directing the child's movements

The PTs direct movements through therapeutic handling in a variety of ways, in accordance with each child's specific needs, the responses from the child, and what the goals are for the session We typically saw different amounts, styles, and outcomes of therapeutic handling across the two observed sessions with each of the PTs. Most prominent in the material is a subtle style of directing the child's movements. A key aspect of this subtleness is the PT's responsiveness to the child's bodily actions and signals, with a withdrawal of directional inputs as the child is able to self-initiate and self-drive the desired play and motor actions. But there were also events when the PTs’ handling appeared to be more intrusive with respect to the direction and timing of the movement they wanted the children to perform. On these occasions, the PTs tended to complete much of the movement for the children rather than waiting for the children to self-initiate and/or control the movement. There are examples in which this handling leads to a child's self-initiated repetition of the same movement, but more often, the result is a child who either quietly accepts being moved or protests against it.

To further clarify these differences in handling style, we first present a sequence with a PT that explains her handling is primarily biomechanical, applying pressure to specific points on the child's body to make it easier for the child to complete desired movements. She is working with 10-month-old David (P7-C12) with motor delay who is trying to roll from supine to prone to reach a toy on the floor placed above his head to the right:

*David is reaching for the toy with his left arm and turns to right side-lying repeatedly but is unable to complete the roll into prone. The PT places her index finger on his left gluteal area and pushes three times in a ventral direction to make him rotate his pelvis to the right, but David resists and flips back onto his back. He rolls again to his right side, and the PT again pushes repeatedly with her index finger. This time her finger is placed on his sacrum. David is still not taking active part in the movement, and eventually she holds on to both sides of his pelvis completes the roll for him*.

The second example shows a more subtle guiding of rolling from supine to prone with 8-month-old Elias (P14-C18) with motor delay:

*Elias is lying in supine on the floor with toys that he wants to reach placed on his left side. The PT guides him from supine to left side lying by lightly pulling his right wrist and arm across his body to the left side and gently pushing the back of his right knee to assist him to rotate his lower body to the left. Elias follows along and as he arrives in side-lying the PT removes her hands. Elias rotates his pelvis further, extends his legs, and reaches for the toys with his right hand. He becomes stuck in a twisted position with little ability to initiate movement against gravity with his upper body. The PT guides him back to side-lying by pushing gently on his right thigh, saying “You can go back”. Elias flexes his hips and knees and activates his abdominal muscles, making him more stable in side-lying, upon which the PT lets go of him again. Shortly thereafter, Elias completes the roll, this time coordinating his muscle activity to work better against gravity and arriving in prone with elbow support on his left*.

As an overall impression of the material, the key to successful bodily interaction with the children is the PTs’ ability to focus their attention on the children's play goal and use their hands and body to assist the children to use their own movement to attain the goal. This sustained attentiveness drives movement that awakens the child's curiosity and drives exploration of new movement possibilities. Although the PT may have to initially introduce and initiate the movements, they gradually become child-initiated and child-driven as the child realizes his or her own new abilities. The best example of such handling is with 18-month-old Felix (P5-C9) who had been cruising and walking with support for some time but has been very reluctant to take independent steps. As stated by the PT: “*When he finally starts to walk, it's gonna be when he feels confident.*” In the observed session, they work on independent standing and stepping embedded in a variety of play activities. We describe three sequences that together tell the story of the PT's subtle and gradual guiding of movements toward his first independent steps:

*In the first sequence, Felix is standing some distance from mom who is holding up a book for him to read. The PT is sitting on the floor behind him. As Felix signals he wants to get closer to the book, the PT asks “Can I help you?”. She places her hands carefully on each side of his pelvis and pushes gently in a lateral direction to help him shift weight, at the same time verbalizing the rhythm “One-two-one-two”, as he takes four guided small steps. As Felix arrives at the book the PT applauds and says “Yeah!”, and Mom comments “Good job. You were pretty willing to let that happen, I’m very proud of you!”*.

*In the second sequence, Felix is standing some distance from mom and a low table with building blocks. The PT is sitting on the floor behind him. Felix has one block in each hand and signals that he wants to bring them to the table, but then takes two small squats as if to go down on all fours. The PTs places her hands on each side of his pelvis, facilitates a tall stance with a light pull upwards as she says “I’ll help you, ready, let's go!”. Again, she facilitates weight shifts and stepping and verbalizes “One-two-one-two”, but this time removes her hands after the first three steps, and he continues the last step on his own. Both mom and the PT cheer and mom says “You didn’t even know it, but you did it!”*.

*The third sequence has a similar set-up as the second one, and the PT again places her hands on each side of Felix's pelvis as he signals that he wants to walk over to mom and the table. This time Felix grasps the PT's left hand and pulls it off his body, with a curious look on his face, upon which she also removes her other hand. He lifts his gaze towards the blocks in mom's hands and takes three independent steps. There is enthusiastic cheering from mom and PT. The PT excitedly states “Those were his first independent steps!” and Mom says “You did it, you took three steps, I’m so proud of you!”*.

During the interview, the PT explained that Felix objected a lot to therapeutic handling over the last few months. Therefore, she was surprised at his willingness to be handled in this session and decided to explore this: “*He seemed more confident and comfortable, I just thought let's go for it!”*

In sum, these results show that to induce a child's self-driven motor actions the PT needs to be subtle, flexible, and precise in the directing of movement during therapeutic handling. Connecting to the enactive perspectives, subtleness and flexibility entail that the PT is responsive to the child's signals in coordination with the child, while precision entails bodily know-how in the placing of hands and direction of pressure to enable the child to actively participate in and eventually self-drive the movement. But the subtleness and flexibility in coordination with the child is not just a bodily coordination; statements like “*you can go back*” and “*can I help you*” indicate that these PTs acknowledge the children's intentions and support their sense-of-agency during the interaction. Thereby, they allow the children to set the premises and use their signals to inform their clinical reasoning of if, when, and how to proceed with their therapeutic handling.

## Discussion

Therapeutic handling that helps the child find good starting positions and provides support and direction of movements when needed to follow play intentions and achieve play goals, enables the child to set the premises in the playful discovery and integration of new movement skills. Turning to the theoretical foundation of this paper, we interpret the scenarios in which the PT emphasized the child's self-initiation and exploration of movements as embodied playing-to-learn-to-move processes that allow the child to be the agent and enactor of his or her sensory-motor play and learning. In contrast to this, the examples in our material in which the child disconnects from play often coincides with the PT's failure to recognize or overruling of the child's intention. We interpret these as situations in which the child's sense-of-agency and enactment of sensory-motor play and learning is diminished or even restricted. In the following discussion, we will elaborate on our interpretations of our findings through the lens of enactive theoretical perspectives before we discuss how these new insights can serve to develop our enactive therapeutic sensory-motor play concept.

An important trait of our findings is that self-initiation and exploration of movements do not require a child's body to be moving on its own. Well-synchronized bodily interactions between the PT and child come with many benefits; they can provide additional dynamic points of support for the child, grade and adapt the level of difficulty of the motor task and reduce the sense of risk as the child takes on new motor challenges—all with the child as the active agent of movement and play. Thus, paraphrasing Adolph and Robinson (18), the child's playing-to-learn-to-move process is embodied, embedded, and enculturated in a dynamic environment that also includes other moving bodies, and the child is learning to move together and coordinate with those moving bodies. Connecting to the phenomenological term mutual incorporation, we interpret these synchronized events as examples of dyadic bodily interplays in which both the PT and child are receptive and responsive to the other's moving body. Given the oscillations during this process of incorporating the other's moving body into one's own bodily perceptions and actions, it comes as no surprise that we find a range of more and less successful events in our material. The therapeutic act of coordinating oneself with rather than with the child requires that the PT actively avoids becoming a unidirectionally leading agent whom the child must follow. Turning to the situations with Adrian in a modified 4-point position and David's rolling from supine to prone, in which the PTs tend to be more direction-specific in their handling and as stated “*too busy with my own doing*,” there is a risk of such unidirectional leading as the PTs focus on their own actions and become less receptive to the children's bodily signals and responses. Consequently, in both these examples, their therapeutic handling leads to a conflict or mismatch with the children's intentions and, as we see in the example with Adrian, a child that is neither moving nor playing.

However, the PT's intentions in terms of therapeutic goals and actions are, and should inevitably be, part of interaction as they serve to unveil new movement possibilities or opportunities or challenges for the child to explore. Coordinating with the child is a matter of utilizing the oscillations between action and reception in interaction with the child to create therapeutically relevant, playful movement challenges. Based on a foundation of receptiveness toward the child's moving body, the PT can choose to take action and provide the child with bodily and verbal movement suggestions and opportunities, that the child can react and act upon. Such utilization of the ongoing oscillating between actions and receptions is what we interpret to take place in the session with 18-month-old Felix, in which the PT repeatedly suggests and assists Felix to take steps, yet with a clear receptiveness to Felix’s response each time. The PT never makes Felix submit to her actions; rather, she receives and reacts to his bodily communication and adjusts her actions accordingly, as Felix progresses from hesitation to resolutely taking his first independent steps. Pivotal to the success of Felix taking his first steps is the subtleness of the PT's handling, along with the withdrawal of handling at the right point in time allowing Felix to become the sole agent of his movement and play. Similar examples are seen in Elias’ rolling from prone to supine at his own will and pace, and with Charlotte standing to reach and grasp the string of beads with the PT's body as dynamic support when needed.

This ability as a therapist to interact with the child's moving body in a way that blends into the background or environment of the ongoing play activity strengthens the child's sense-of-agency as it enables the child to stay engaged and focused on the play goal, move without hesitation, and willingly struggle with the motor tasks that the play activity demands. This is contrasted by the examples in which the PTs’ handling seems to enter the foreground of the children's attention because it does not align with the children's own movement and play initiatives, interactional breakdowns more frequently occur, and the PTs’ handling is rejected or counteracted by the children. The examples with Adrian and Beatrice in 4-point kneeling highlight a mismatch between the PTs’ goals and the children's current abilities and intentions. If the PT reflects on the result of the handling and acts upon this disconnect the relationship can be repaired and play can continue, as we see in the example with Beatrice playing with her light-move-and-sound-toy. But in the example with Adrian, when the PT admits that she fails to discover this disconnect, the result is a stressful spiral with increasing levels of objections from the child, and a PT with an increased focus on therapeutic actions and lowered awareness of the child's play initiatives. Along our theoretical scales, one might say that coordination and or incorporation dissolves, and neither of them gets to follow their intentions or maintain a sense-of-agency.

This example underscores that a child's attention and dis/engagement in play due to the PT's handling is a matter of enabling the child to become the agent of play, with a sense-of-agency or being in charge of the movements of his or her body. While this sense-of-agency may well include the willingness to move along with the PT's therapeutic handling, it always includes the opportunity to choose otherwise, as confirmed by the PTs’ verbalizations such as “*You can go back*” and “*Can I help you?*”. This building of the child's sense-of-agency can also be understood as the foundation for the PTs’ embodied knowledge and decision-making about when it is time to withdraw themselves from bodily interaction. The three PTs in Elias’ rolling from prone to supine, Charlotte's standing to reach and grasp the string of beads, and Felix’s practice of independent stepping, were all receptive and perceived the bodily signals and behaviors indicating these children were ready to be fully in charge for their bodily actions during play. These PTs decided to let the child take on this new challenge of moving without support or guidance. Moreover, they were able to continue their coordination with the children and scaffold the children's success and sense-of-agency in reaching their sensory-motor play goals. This is best seen in the example with Charlotte reaching for the string of beads, in which the PT coordinates herself in a readiness to support the child that promotes Charlotte's continued trial, error, and eventual success in her sensory-motor play endeavor.

The final key finding in our material is the PTs’ and children's mutual attention to the play activity and goal. This facilitated the children's engagement and willingness to explore new movement possibilities. This corresponds with our findings in the paper that introduced the enactive therapeutic sensory-motor play concept (13), in which the establishment of shared intentions is highlighted as a vehicle for new and enriching learning experiences. In this current study, three of the examples show a similar merging of the PTs' therapeutic handling and the children's play intentions. In the example with Beatrice in 4-point kneeling, the PT's intention of training in this position is merged with Beatrice's eagerness to play as a driving force, and Beatrice's new ability to take some support on one arm and reach and play with the other is the result. Although one might question the relevance of this rather advanced position for a child with severe motor impairments, the choice of a favorite toy awakens Beatrice's play engagement and enables her experience of sense-of-agency as being one that causes the light, movements, and sounds to appear. The PT's ease in helping her return to weight-bearing on her hand the second time around also indicates a potential that she can learn to play in this position.

With Charlotte standing and playing with the string of beads, her play engagement guides the developing bodily interactions between the PT and child, leading them into the mutual exploration of a dynamic standing position with Charlotte actively supporting her own weight in an upright position. Noteworthy, as explained by the PT during the interview, the situation spontaneously evolved and “*just came naturally*.” This can be understood as embodied enactive clinical reasoning-in-interaction (14), in which her bodily interactions with Charlotte brought her to suddenly find herself in this position. Her bodily know-how of interaction with Charlotte preceded her conscious awareness of what her therapeutic choices of action should or should not be. In hindsight, she realizes that their bodily interaction and her therapeutic choices facilitated Charlotte's active movement explorations and play engagements, and along with these came therapeutic windows of opportunity to work on standing and reaching to play. As such, this example shows a new dimension when it comes to the enactive understanding of coordination and sharing of intentions; the interaction process can gain a momentum of its own that neither of the interactors can control (1, 13, 22). Along with this momentum, in coordination with each other, emerge new therapeutic possibilities that were not planned.

In the example with Felix who is learning to take independent steps, he is initially engaged in playing with the book and the blocks. While these play engagements are at the front stage of his attention, he seems to be aware these games can be merged with the PT's intention—or second agenda—of wanting him to take independent steps. As the session proceeds, he gradually includes the PT's intention into that of his own, and the initial guided stepping experiences transform his long-standing reluctance into curiosity and a new confidence that eventually makes him take the initiative to release himself from the PT's hands and explore the possibility of independent stepping. It is a self-initiated, exploratory, intended motor action and learning incorporated into his play, and perhaps even a playing-to-learn-to-move event in which the skill of walking becomes a play activity itself. The PT confirms that this was a result of the momentum of interaction taking the lead through her statement: “*He seemed more confident and comfortable, I just thought let*'s *go for it!”*

In conclusion, our data material demonstrates fluctuations of therapeutic handling that move along the scale from unilateral and mutual incorporation, also understood as variations along a scale of coordination to and with each other. There are examples in the material with therapeutic handling that it predominantly unilateral coordination to the child, restricts the child's sensory-motor play, and diminishes the child's sense-of-agency. But we want to emphasize the richness of examples in which mutually incorporated therapeutic handling in coordination with the child enriches the child's playing-to-learn-to-move process by providing novelty and facilitating the child's sense-of-agency in the self-initiated discovery, exploration, and refinement of movement possibilities. Returning to the enactive therapeutic sensory-motor play concept, this current study supports the view that therapeutic handling can be a means by which children discover and explore new sensory-motor movement and play possibilities. More specifically, our data material shows that the PT's handling approach needs to preserve the children's sense-of-agency by being subtle, flexible, and responsive to the child's play initiatives. Essential to this is the PTs’ attentiveness and support of the children's engagement, self-driven action, and problem-solving of tasks. In the PTs’ effort to merge therapeutic handling with children's play and movement initiatives, allowing for the momentum of interaction and its powerful yet unpredictable forces to unfold can open therapeutic windows that provide both the child and PT with new experiences and learning opportunities.

Finally, this exploration of therapeutic handling shows that we need to move beyond the dichotomizing debate of hands-on vs. hands-off approaches. Connecting to the overarching theme of therapeutic touch, therapeutic handling as a hallmark in physical therapy requires an awareness during bodily actions and interactions and a mutual understanding of how these can become therapeutic, supportive, and guiding acts that support the patients’ enactment of their world.

## Data Availability

The datasets presented in this article are not readily available because it was not possible to anonymize data, but extracts are provided as **Supplementary Material**. Requests to access the datasets should be directed to ragnhild.hakstad@uit.no.
