# Supplementary material for: Enactive explorations of children's sensory-motor play and therapeutic handling in physical therapy
Source: Front Rehabil Sci. 2022 Oct 11;3:994804. doi: 10.3389/fresc.2022.994804 (PMC9592846; doi:10.3389/fresc.2022.994804)
Supplement: Supplementary file 2 [file Presentation1.pdf]

Therapeutic handling - overview of included sessions

P1-C2

Boy, 4m. Session at PT office with a main focus on improving posture and head control in prone, but also grasping toys in supine and rolling from supine to prone. Overall, the boy is most interested in social play, he responds quickly with smiles and sounds to both PT and mom. They start out in supine, and the PT introduces toys in his visual field for him to grasp. He looks briefly at it and grasps it for a short while with his left hand when the PT brings the toy to his hand, she also tries to support him over his chest to make grasping easier, without getting any results from that either. Next, the PT tries to facilitate the boy's rolling from supine to prone, mostly by making approximations over his shoulder and pelvis in side-lying. But there is little active participation from the boy, and she ends up rolling him passively over. In the prone position, mom presents toys for him to look at, while the PT tries out different ways to position him and facilitate his head lifting. But the boy is lying passively and chewing on his hands. Multiple times, the PT returns him to side-lying and works with approximations again, then back to prone and continues her attempts to make him work against gravity. Notably, his most prolonged head lifting happens when the PT is not hands-on trying to help. At the end of the session, the PT holds him in half-sitting and works with him holding his head in midline. But it is difficult because his head either falls forward or he pushes back in extension.

During the interview the PT explains that she is struggling to figure out how to use her hands and tries out techniques that she has recently learned, but without much success. Although she does perceive that he shows more active work against gravity/ head-lifting action towards the end of the session, most likely due to him gradually becoming more awake and active.

Thoughts on therapeutic handling and merging with play

At an overall level there is little play in this session. Although he looks at the toys presented to him, they don't seem to wake much engagement. One might wonder if the PT's frequent handling/ changing disturbs him more than it helps him play. It may also be that that play activities are too advanced for this boy's capacity and or interest.

P3-C5

Boy, 1y6m. Session in kindergarten with kindergarten aid and mom present. Emphasis on rise to stand/ standing/ cruising. PT sets up room with different standing heights in a U shape. The kindergarten aid has the role of play partner, and mom is primarily observing.

As the PT tries to assist the boy in pull to stand, he protests and calls out for mom. Next when the PT tries to facilitate a better knee stretch, he quickly protests again. As a response, the aid "distracts him" back into play and the PT withdraws his hands, commenting that he is doing such a good job by himself, so it is better to keep his hands off. Next, they work on sideways stepping while playing with balls. The boy squats as he tries to move sideways, and the PT guides him into a half-kneeling position. When they work their way up again it is still with a lot of protest. Mom comments he doesn't complain like this when moving around at home. They continue to work on sideways stepping, the boy still protests but at the same time tries to take steps and reach the balls. The PT now

steps the boy's feet sideways for him, the boy protests shortly each time, but is quickly re-engaged with playing with the balls, putting them into a container and throwing them at the floor.

After a short break they work on standing again, the boy continues to complain both while getting up and during stance. The PT asks if he wants to get down, and guides him down to knee standing, also meeting protests from the boy. They continue all the way down into side-lying and the boy calms down. The PT tries to facilitate some rolling but stops again due to protests from the boy. The PT turns to mom while the aid continues playing with the boy. At this point the boy quickly and without protests gets into a kneeling position by a lower pillow. Again, this time while moving on his own, he starts complaining but still continues to crawl between heights, gets up to knee standing and down again, and engages himself in play in between his complaints. Towards the end he is also increasingly eager to chase items that are placed out of reach, and his objections are decreasing. They are then able to complete a new longer standing session with engaging ball play between the boy and the aid, during which he takes an independent sideways step.

During the interview the PT explains that this boy has been protesting a lot during therapy, and that today's session is one of the better ones. The PT explains that she acknowledges his objections, but also wants to show the boy that is can be a good thing to work through some struggle together. She perceives that they are in a process of building trust and accomplish more together every time they meet.

Thoughts on therapeutic handling and merging with play

There can be several reasons for the boy's; are the tasks too difficult? Is the PT's handling too fast for him to move along with? Is there a sense of losing control or being disturbed in his own way of moving/ problem solving how to move? Also, it is hard to judge their relationship; are the objections a result of a PT that has pushed this boy too far too many times before, or a building of trust that is moving in the right direction? Noteworthy, the boy transitions quickly between play and protest, could it simply be a way for him to communicate hard effort and struggles, in his discovery of new motor opportunities? His increasing eagerness and willingness to move indicates that he does have a positive, motivated play-and-learning curve through this session.

P3-C6

Boy, 8m. Session at PT's office, mom is present. They start out working in prone, the PT presents toys that the boy is eagerly trying to reach, but they are too far away. The PT tries to help him support weight on his elbows, next provides a bolster under his chest for support, but then removing it again after a short while and returns to give manual support on one of the elbows and over his pelvis, repeating the exercise on both sides. The boy is working actively to support himself and eagerly engaged with reaching for the toys. The PT is attentive to the boy's engagement, but at the same time attentive to the details of the boy's motor performance. Next, the PT guides the boy up to a modified 4-point support position with knees flexed, buttocks to heels and elbows on a bolster. She places a mirror in front of him, commenting that it was very successful last time they met. The boy looks at his reflection and also tries to reach for the mirror, but then the PT pulls his arm back because he wants the boy to use his elbows for support. They struggle for a while before the PT decides to discontinue this exercise and move down in prone again. The boy explores the mirror with both his hands and his mouth, but then the mirror is quickly removed, and the PT moves on to an exercise from sidelying to sitting and back down again, with therapeutic handling to support and guide the boy's movements. The boy complains but calms down again when the exercise is over.

The PT is now moving more quickly from task to task, guiding the boy back into the modified 4-point-position in front of the mirror, next placing the boy in supine and rolling him over into prone and work on elbow support. The boy is complaining and is given a short break in supine, then guided to roll back into prone, then a couple of more rounds from sidelying and over to prone again. The boy is now getting more frustrated, he seems to still be interested in a toy nearby but too frustrated to ‘collect himself’ and reach for it. The PT turns the boy into supine and presents the toy for him to grasp. But the boy will still not grasp it, he is lying with his hands down to the sides and babbling to the PT for a short while. The PT then moves him back to the side position to work up to sitting again, and also on the rolling from side to prone. The boy is now very frustrated and crying, looking occasionally at the toy but frequently disrupted by new positioning moves from the PT. The PT continues to position him into new ‘exercises’ and the boy is now crying continuously. To round off the session in a good mood, the PT returns to the mirror activity in prone. The boy calms down but is frustrated when the PT again moves him out of position.

During the interview, the PT explained that the previous session they had ended with a lot of crying and was relieved that today’s session worked better. The PT usually performs more Vojta techniques during sessions with this boy but decided to leave them out today due to the researcher’s visit and the purpose of the study.

Thoughts on therapeutic handling and merging with play

During this session, toys are only used to provide training situations, with very little attention from the PT on how to support the boy’s play engagement. Starting out with a more ‘tuned-in’ approach, the PT’s pace gradually picks up as the session proceeds, with an increased focus on repetition of training exercises, and decreased attention to the boy’s expressions of disengagement and distress. As the boy’s frustration levels increase, the PT talks to him and confirms that she understands that this is stressful but does not change her course of action to accommodate the boy’s expressions and needs. Overall, exercises and repetitions seem to be more important than the boy’s ability to initiate and engage in play.

P5-C9

Boy, 18m. Session in family home, with mom present. Focus on standing and initial stepping skills. They start out in standing with a push-car, reading a book. The PT moves the push-car out of reach, and the boy is standing alone with a wide stance and able to grab and throw a ball in interaction with mom and the PT. The PT then sets up a ball tower to play with on the coffee table, and both the PT and mom try to tempt him to stand up there and play. But the boy hesitates and finds objects of interest on the floor instead. The boy points to a stacking tower, the PT follows up on his initiative and they start stacking and tearing it town together. The PT again tries to tempt him over to the coffee table, but he still does not want to get up. Next the PT introduces a shape sorting box and is able to bring the boy up to stand and have him put the shapes into the box and squat down to pick up new shapes. Then he goes down into crawling position and picks up a book for mom to read. Mom holds the book up and helps him come back up to stand, and the PT gently guides him in moving his feet to a narrower stance. The PT asks mom to move backward and see if he will make a small step. He reaches out with his arm to turn the pages but will not move his feet. The PT asks if she can help, and eventually is able to help him move a few steps toward the table by facilitating weight shifts. Mom moves away to see if he will step towards her, but he is able to find support on furniture and squats down to get to her instead.

Mom then pulls out a bag of big blocks, and they want him to walk across the floor to build with her by the pouffe. The boy is eager to build and tries to let go off his support surface, 'I'll help you', says the PT, and gently puts her hands on his pelvis for support and facilitating steps as she says: 'one-two-one-two'. He accepts her hands on his body and walks a few steps, pauses, and then continues the last few steps towards mom. Along the way, the PT removes her hands, and he takes his first little step on his own. Mom and the PT cheers, but the boy seems unaware of what just happened and is focused on building with the blocks. They arrange a second try, the boy has a curious facial expression as the PT starts facilitating again, they take a couple of steps together, she then quickly removes her hands, and he continues on his own for the next 2-3 steps. There is plenty of cheering from both mom and PT, at the PT says: 'those were his first independent steps!'. But the boy is again busy with the blocks. Mom comments 'he's like, yeah big deal, I'm gonna build these blocks'. Onwards, both mom and the PT are eager to repeat the success, but he is less willing to let go of his supports, less willing to stay on his feet and keeps squatting instead of taking steps. Eventually in the last attempt he again takes a few steps facilitated by the PT, but then closes his eyes and sits down and the session is over.

During the interview, the PT explains that this boy has not allowed much handling over the last few months, so she was surprised of his willingness to be handled today and decided to explore this window of opportunity and see if he was ready to try stepping. She confirms that she through her handling aimed to support his natural moving patterns, follow his lead and allow for him to be in charge.

Thoughts on therapeutic handling and merging with play

This is a very playful session, but at the same time with a clear therapeutic focus on standing and stepping. There is a natural flow between activities, the boy gets to follow his own initiatives, but sometimes mom or the PT make suggestions that provide new training situations for standing and stepping. The boy prefers to use his established motor repertoire and move by himself, but also shows a playful curiosity toward taking independent steps, and seems to enjoy the experience of being successfully facilitated by the PT. But at the same he clearly signals that he first and foremost wants to play, and also seems to reach exhaustion after challenging himself to take his first independent steps.

P7-C12

Boy, 10m. Session at home with mom present. They start out on the floor, the PT asks about him rolling, grabs the toy that he is playing with and places it on his right side to see if she can make him roll over. He is only able to come over into sidelying, and the PT uses her fingertips to give a slight push on his pelvis to direct his movements onwards to prone. Once he has rolled, she also helps him pull out his right arm, and mom comments that he still has problems doing that by himself. He turns himself back into supine after a short while, and the PT moves the toy over on his left and facilitates him rolling to prone by pushing slightly on his pelvis and at the same time pulling his right knee into extension at the end of his rolling sequence: "I made you do it, I know", she says. He rolls back onto his side and is quickly back in supine again.

Next, mom tries to make him pull-to-sit, but he does not want to come up and wiggles around instead. The PT switches to working on moving from side-lying to sitting and recommends mom can work on this. She also works on the rolling again and has him staying in prone for a longer period of time. He is very motivated to reach a toy and once he gets it the PT applauds and 'awards' him by allowing him to roll back into supine with the toy in his hands. Mom now tells that the boy is in fact

able to sequentially roll across the floor. ‘Oh, so you can roll’, says the PT, and again tries to make him roll over his left side. Now there is a longer sequence of rolling to both sides, with the PT fetching the toy repeatedly, placing the toy on the floor and providing guidance over the pelvis to help him complete the roll. At this point, the boy’s complaints increase, and mom says that he is probably getting tired.

During the interview, the PT explains that this boy has been happy observing his surroundings and it is only the last couple of months that he has shown interest in moving by himself. She trusts mom’s information that he can roll even though we didn’t get to see it during the session. The PT says that she uses bio-mechanical principles in her reasoning of where to place her hands and how to facilitate a child’s movements, but like we see in this session, she doesn’t always reach her goal.

Thoughts on therapeutic handling and merging with play

Overall, this PT is taking an observatory stance and there is little social interaction as part of their play. Play thus primarily serves as a tool for observing or stimulating specific motor performances. Still, the PT is attentive to his signs of engagement and interest, picks up on his initiatives and uses them to make him work with rolling and prone positioning. But whenever she wants to get see any specific motor function or get any specific action going, she tends to remove items from his hands or place new items in his hands without giving notice. However, the boy seems to accept toys being taken away from him and easily engages in new items that are introduced to him.

P8-C15

Girl, 2y. Session at home with granddad present. They start out sitting on the floor with straight legs, PT sitting behind the girl to provide support. They have a driving wheel toy with lights/ sounds in front of the girl, the PT is talking to grandfather and at the same time helping the girl in her handling of the toy, but with little visual monitoring of what she is doing. The PT gradually tries out different sitting and kneeling positions and end up in left sided “mermaid” sitting with support from the PT, where she can handle the toy with her right hand.

The PT moves on to try a straddling position on her lap, providing support to the girl’s upper body. The girl is complaining, and the PT puts her down on the floor to try some rolling. They end up in right side-lying where she handles the steering wheel toy with her left hand. The PT helps her up on elbow support which is difficult at first but after being held there for a little while she starts to move both her upper arm and her head more freely and is able to push one of the buttons on the toy.

After introducing the girl’s favorite light-move-and-sound toy, the PT helps the girl over into prone and places her arms for elbow support. The girl spontaneously takes support on her right elbow and with the PT’s facilitation also briefly with the left. Next the PT tries to get the girl into 4-point kneeling, but she extends all the way up in knee-standing, and the PT uses a lot of handling to get her back down again and help her take support on her left hand to play with her right. The girl complains but they are able to get into position with the girl eagerly reaching and touching the toy. Next the PT moves her over into left side-lying with elbow support with the steering wheel in front. The PT sings ‘The-wheels-on-the-bus’ and moves the girl’s right arm to gesticulate the song moves, but it is hard to tell if the girl is engaged by this at all. The rest of the session is spent testing out her standing device.

During the interview the PT explains that this girl is difficult to engage in play and that she needs to give ‘maximal assistance’ to provide support and help the girl find ways to play with toys of interest.

Thoughts on therapeutic handling and merging with play

This girl has very limited communication abilities but shows engagement and drive to play with her toys, and moans/ grunts when she is not happy with what is happening. The PT is attentive of her signals and changes her actions according to them, but in not always attentive to maintaining and reinforcing play situations that appear to be engaging the girl. They go through a large range of positions and skills during the sessions. Are they all important for the PT to go through, or is the PT struggling to find optimal strategies and therefore continues to move on to new things? Either way, the result are that there are only short glimpses of play engagement during the session, and these glimpses are often interrupted by new positionings / handling/ removal or moving of toys.

P9-C16

Girl, 11m. Session at PT's office, with mom and sibling present. The PT sits the girl down on the floor with legs straight and a xylophone to play with. While the girl handles the toy, the PT tries to position her legs and pelvis. The PT places a wedged pillow under the girl, which makes the girl sit up straighter. Next, the PT puts a sock on the girl's right hand and presents a box with different objects. The girl tries to pick them up with her right hand and gradually becomes more active with the left as well. One of the objects has a string to pull at, the girl finds it highly engaging and pulls at it several times with her left hand. The PT helps her hold the object with her right hand, and also supports/ straightens her left leg. Now the PT picks the girl up on her lap, tries to make her stand but ends up sitting her back down on the wedge again, stretches her left leg/ ankle and provides support on the girl's pelvis, but also removes her hands to observe her independent sitting.

Next the PT picks the girl up again and places her in prone. The PT starts stacking cups on a low firm foam pillow, the girl show interest for a moment, but grabs on to a ball nearby and also crawls to a toy behind her instead. When she returns to the pillow the PT tries to help her climb onto it, but the girl resists and the PT lets her return to the floor. The PT suggests they can try standing. She arranges a stack of firm foam pillows for her to stand and lean into, mom sitting behind the girl and holding onto the girl's pelvis while the PT tries to make her reach and grasp with her arm. Mom comments that it's always hard to tell how much support she should give, but that she recognizes the same pattern as in sitting, that the position of her arm and trunk indicate how good her seating position really is.

After some transitions via mom and the floor the PT the girl on her lap and puts the girl's pantyhose back on, realizing that the girl is actively helping with her right foot. As she goes to pull the pantyhose all the way up, she rises the girl to stand and raises herself up to knee standing to provide support for the girl. In this position she makes the girl reach and grasp a string of beads with her left hand and they play with variations of this activity for a while. They sit halfway back down again, and as the girl slides all the way down in sitting before the PT picks her back up on her lap.

The PT now places the girl back down into prone, the girl crawls around and picks up objects and 'checks in' with mom with cheerful babbling. The PT asks if they ever see her up on all four, mom says no, and asks if this is something they should work on. The PT confirms that they can try it out, places her in 4-point and demonstrates how they can support her in the position. The girl is still busy handling different objects and becomes frustrated with the PT's handling disturbances. Next the PT sits the girl up on the floor and supports her while she is handling and mouthing toys. She also explains to mom how she provides support from behind and pushes a bit forward on the left side, and that she needs this to sit straight. After some conversation, the PT puts the girl down in prone again, moves and stretches her left leg back and forth and tries once again to get her up to 4-point position.

But the girl protests and the the PT backs off. After the camera is switched off and they are about to leave, the girl crawls a longer distance across the room and out into the hallway.

During the interview the PT explains that she was surprised at her own 'choice' of working in a standing position with such high postural demands, but we agree that the video clip shows that the girl handles it well and is working productively against gravity and simultaneously using her left arm.

Thoughts on therapeutic handling and merging with play

This PT is attentive to the girl's play engagements, allows her to follow her own leads and follows up on the girl's interests when making new activity suggestions. There is a 'laidback' mood with much time spent on observation/ gathering information, but this is interspersed with goal-directed play activities that is engaging for the child and at the same time challenges her use of the left arm for reaching and grasping, 4-point kneeling and standing, with handling that provides sufficient support and helps the girl work against gravity. Occasional mismatches in which the girl complains or disengages are quickly repaired, and the girl maintains her exploratory play engagement throughout the session.

P9-C17

Boy 7m (5m CA). Session at PT's office, both parents present. The PT places the boy on a wedged pillow on the bench and introduces a toy. The boy immediately looks at it, rotates his head to the left and grasps and brings it to his mouth. The PT retrieves the toy and presents it again on his right side and facilitates him rolling to right sidelying and over into prone a couple of times. After a short while in prone the PT rolls him back to sidelying so that he can handle the toy for a moment. The PT presents him a new toy, the boy traces is while the PT rolls him to back to sidelying and into prone again. After a short while the boy complains, the PT turns him back into supine and lets him play with the toy. Next, she presents him his feet, holds them in position as he grabs them with both hands and plays around with his fingers on them, looking with a focused stare at his own performance.

She tries again to put him in prone but picks him back up when he again complains. She comments that he seems tired and that they are almost done, and after a couple more attempt at playing they end the session due to his complaints. During the interview the PT explains that due to medical issues it was difficult to work with him today.

Thoughts on therapeutic handling and merging with play

The boy seems to be very 'play oriented and driven', both when it comes to exploring toys and engaging in social interactions with the parents and the PT. They are all very attentive at his signs of interest and engagement, and the parents involve themselves in the activities either on their own initiatives or when encouraged by the PT. But overall, this session does not have much room for play, and the PT seems to be struggling to find good strategies for how to provide him with motor challenges without stressing him.

P10-C18+19

Session with two boys, 2y. Both mothers and one father present, plus teacher and OT who are in charge of activities, while the PT's role is to help the boys in their motor actions. After entering the room, both boys are placed in sitting on a low 'bench' build out of gymnastic mats, and they sing songs together. Next, they move on to play with magnetic building blocks in standing. The PT helps

BoyII obtain kneeling position and facilitates him in moving up to stand. Now the boys are standing side-by-side but playing individually with the blocks. BoyI steps over to the next bench and for a short while the PT is 'multitasking', trying to provide support for both boys when needed. Then OT steps in to support BoyI, and PT can focus on facilitating sideways stepping with BoyII.

Suddenly, BoyI moves towards the slide at the other end of the room, and the PT picks up BoyII and carries him over there too. BoyII needs more assistance than BoyI, but they both get to do a couple of rounds on the slide. Next, mom of boyII places him in a ball binge instead and he calls out for boyI to join him. BoyI crawls up the step to it and hangs on the side and reaches his hands down to the balls. He crawls further, and with minimal support from the PT he problem solves turning around and lowering himself into the binge. After this, they seat the boys in chairs and blow bubbles with them.

During the interview, the PT confirms that it is challenging to work with both children at once and also that the group of people working together can sometimes restrict her in following up on things that she as a PT might want to pursue. Still, the value of creating a peer-play environment outweighs these challenges and creates a good arena for them both to challenge their motor skills and learn new things.

Thoughts on therapeutic handling and merging with play

This session is the only 'group' setting in the material. The boys start out as more 'individually' driven and seem to connect more with each other as the session proceeds. But they both require individual follow-up from both staff and parents, and the PT has to jump in and out of situations with both boys to provide them with support and facilitate movements when needed.

P10-C20

Boy, 2y. Session at PT's office, mom, grandmom, kindergarten aid, teacher (only observing) and OT present. The boy starts out on the slide, where crawls down on all four with help from the PT. The PT suggests they go over to the whiteboard, the PT helps him up to stand and guide him over some obstacles on the floor. She then guides him back down on his knees and starts a tractor over the floor to make him crawl towards the whiteboard. He crawls along, seeking out obstacles on his way that he wants to crawl over on his way to the whiteboard. Here the boy is sitting on the floor and finds magnetic figures that the PT/OT puts up on the board. He finds a marker to draw with and wants to get up and draw on the board. There is a railing below the board, the PT assists him in pulling up to it and supports his pelvis in standing while he draws. He switches between sitting on the PT's lap and pulling up to stand as he continues drawing. At one point he stands up quickly and self-driven, impressing the PT and OT; 'that was fast, straight up' they say. Next, he comes all the way down to the floor to get a new marker and wants to get back up to the board. The PT assists him in standing up from his kneeling position. He stands for quite some time with support over his pelvis from the PT.

Next, they play with a tractor on the floor, the PT and OT arrange a new training site with a bench in standing height and bring the tractor over to it. The boy crawls over and pushes himself to stand with guidance from the PT. The OT starts playing with the bus, and drives it across the bench, and the boy is told that he can walk over to get it. He tries to take a few steps, but then sits down by leaning backwards in an uncontrolled manner but is secured by the PT controlling him down. The PT then helps him back up to stand, and he plays with the people inside the bus. One of them falls on the floor, and the PT guides him back down on the floor to get it. He quickly crawls to get the missing person and returns to the bench again. The PT assists him in pulling back up to stand. The OT moves

the bus to the other side again, and the PT tries to help him cruise over to it. But he is not very willing to take steps, the PT tries to verbally instruct him but without results. He then drops a person again, the PT again verbally explains how he should get down, and simultaneously guides him physically. He wants to get back up, and nods when the PT asks if he wants help, and after a little chat back and forth about him ‘working’, he comes back up. The PT places a squared pillow behind him and informs him that it is there to sit down on. They play for several minutes putting people in and out of the bus, and the OT places some of them on the floor so that he must sit down on the pillow to fetch them.

After a short sequence on a trampoline, they move to a walkway across the room. The PT helps him take a few steps, and he holds on to the railing with his left hand and simultaneously plays with a ball with his right. The PT says ‘can you try to have your legs in a better position’ and goes to move one of his feet for him. This startles him and brings him out of balance, and he drops the ball. Now the OT brings the ball to the other end of the walkway and the PT supports his trunk while he holds on to the rails and steps forward. But he is leaning backwards as he walks and eventually falls down on the PT’s lap. He comes back up to stand again, sneaks his head under the bar and crawls towards mom.

During the interview, the PT explains that the boy she needs to provide a lot of support to this boy because he is unpredictable in his movements and does not seem to plan ahead much. But he also surprises them with timing and precision of movements at times.

Thoughts on therapeutic handling and merging with play

This is a very social and playful boy in a session that is play driven; the boy gets response from the group of adults on the initiatives that he makes, he gets to shape the content of the session and uses his communicative/ social resources to create a play environment for himself. He is easy to engage in new play activities, and the PT is in ‘his background’ and supports him in his bodily efforts as he works to complete the actions that he wants to do. In some instances, however, the PT’s effort to improve posture or chance his positionings are disturbing his play rather than supporting them. But these instances are quickly repaired, and they are able to continue with the same or new activities.

P11-C21

Girl, 2y. Session in family home, grandmom is in the home but not involved in session. They start out with ankle stretching before putting on her AFO and shoes. Now they are ready to do a large puzzle that the girl enjoys. The PT places the box on the floor and creates stepping obstacles out of her legs that the girl must cross for every piece that she’s picking up. Both the girl and the PT are verbalizing how she should move; ‘left foot’, ‘keep your knee up’, ‘big step’. Next the PT advances the activity by hiding pieces under the coach. The girl is very accepting of these specifications and also allows for the PT to guide her movements of her legs in taking long steps, holding the knee straight while squatting etc.

Half way through the puzzle the girl seems to be getting tired and is complaining more. The PT suggest they hide pieces in the stairs, and after a bit of negotiation the girl agrees. The PT guides her walking in the stairs, the first time without complaints but the second time with more of a resistance from the girl. The PT wants to guide her movements and asks her to use her right hand for support, but the girl complains and so she gets to do it ‘her own way’, supporting herself with her left hand instead of right. Next the girl starts to sob and collapses in the stairs. The PT suggests new activities that they can do after cleaning up the puzzle, the girl first agrees but then says no and ends up having a complete meltdown. When the PT suggests they can color, she turns around and says ok, and all of

a sudden, she's back on track. The PT places a bucket under her left foot, at first accepted by the girl, but then after a short while she says 'no, no standing'. The PT removes the bucket but brings it back shortly thereafter without any objections from the girl. They continue to draw and the girl standing on her right leg and supporting herself with the right hand on the table and drawing with the left. As the girl brings her foot down a couple of times, the PT allows for the break but gets her foot back up on the bucket again shortly thereafter.

During the interview, the PT explains that she has spent time building trust with this girl and has struggled to find activities that she enjoys doing. But she feels that they are now on a good track and so the meltdown in today's session was unusual for her.

Thoughts on therapeutic handling and merging with play

This girl has a particular way of playing, very structured and organized and with very advanced verbal skills. The PT catches well onto the girl's way of playing and they seem to have developed a predictable routine of activities that works for them. In it, the PT embeds a range of specific motor challenges addressing balancing, squatting, transitioning to the floor and up and use of both hands. By this, specific actions and therapeutic handling that challenges the girl's strength and flexibility and facilitates a more varied motor behavior are accepted by the child.

P11-C22

Girl, 12m. Session in family home with grandmom present. They start out in sitting on the floor, with kitchen appliances as toys. The girl seeks down to prone several times, but the PT facilitates her coming back up via side-lying. All the while she tries to engage the girl in mutual play and exploration with the bowl and ladle, the girl is interested in brief moments, but is overall mostly engaged in solitary object exploration.

Next the PT picks the girl up and sits her in front of the coach and the girl initiates pull up to stand. The PT immediately handles her to ensure that she puts her right leg forward as she pulls to stand. The girl seeks down to the floor and they get a couple of rise-to-stand repetitions in before the girl crawls away. The PT picks her up and returns her to the coach, and over time tries to maintain a standing/ squatting and playing activity by the coach. After a while of playing in standing, the PT initiates walking training with the girl, by holding both her hands and leading her across the floor, saying: 'da-de-da-de' as they go. The girl walks along and mimics the sounds of the PT. After a break in sitting, they return to cruising by the coach, this time the PT places her in standing, and the girl quickly starts stepping sideways, first to the right and then to the left. Then there is a pause where the girl comes down to 4-point kneeling. The PT decides to switch activity again and pulls out a zipped bag of plastic horses and sits the girl down. The PT opens the bag for her and pulls out some horses to play with. The girl is eagerly reaching for the horses and tries to lay down on the floor to play with them. The PT gently handles her to stop her from laying down/ helps her back up into sitting. The girl keeps pushing back to lie down, put the PT supports her back and legs to make her stay in sitting and playing.

In the end the PT picks the girl up and sits her on the coach and starts singing 'head-shoulder-knees-and-toes'. The girl immediately focuses in on the PT's face, hums along and gestures with her hands on her head. This singing session shows a very different level of connect between the PT and child than what is previously observed during the session (where the girl seems to be more 'in her own world').

During the interview, the PT explains that she was focusing on providing the girl with varied sitting and standing experiences, and that subtle guidance worked well to encourage her to stay in positions over time. She confirms that it can be difficult to understand the girl's play engagements but that she tries to introduce different activities and follow the girl's lead as best she can.

Thoughts on therapeutic handling and merging with play

This girl is playing, but with little social interaction with the PT. The PT tries to mimic her sounds and verbalize what they are doing together, but the girl shows very few signs of awareness to this. Despite this, the PT continues to make attempts at social play in terms of mimicking, peek-a-boo, but also allows for the girl to play in her own way. Noteworthy, whenever singing is introduced, the girl becomes more focused with her gaze and also seems to have more purposeful vocalizations in sync with the PT. Likewise, the PT's 'da-de'-s as rhythm words to accompany walking seems to catch the girl's interest and instigate new motor possibilities. Overall, the PT is able to bring this girl to 'her next skill levels' in terms of sitting and standing to play, instead of her own preference which seems to be down on the floor. The PT's gentle way of leading her into new positions seems to be an effective way to help her discover new movement opportunities.

P12-C23

Boy 1y6m. Session in family home, both parents and two older siblings present, but not involved during session. They start out with the boy stepping one foot onto a low stool and reaching for snacks in a bowl. But the boy is not very interested, and the PT instead gives him a light-up magic wand and puts in on a therapy ball in prone, so he can reach and bang the wand on a box in front of him. He is briefly engaged but mostly wiggles and grunts in dismay. The PT tries to keep the activity going with different objects, and also handling him so that he can transition on the ball between prone, side lying and sitting. However, there are very few child-initiated movements. She also tries to interpret what his interests and requests are given his lack of words and emphasizes single words that are salient to the situation (e.g., more). The boy cooperates for a little while, but then starts to complain, reaches for his dad and says no to new suggestions from the PT. When dad leaves the room, the boy reconnects with the PT and they start playing with rubber animals, while he is sitting on the therapy ball and the PT is handling him in different directions that challenges his balance and increases his trunk rotation. She invites him to use force in different ways – pull, squeeze, and throw the toys – which he does.

After a failed attempt at having the boy help clean up toys, the PT places him on her lap and hand-leads him to complete the clean-up with him still objecting to the task. She then pulls out stickers, and he quiets down and is interested in the new activity of putting stickers on a piece of paper on the low stool. The PT handles him so that he must stand with most of his weight on one foot and lean forward with support on one hand and stick with the other. The boy completes a couple of stickers before he complains and bends his head down. The PT asks if he is done, and has him help clean up the stickers, which he does without complaints. Next, he gets a short snack break, the PT hides some snacks inside the stepping stool, and the boy moves over to investigate it. They hide the entire snack-bowl, the boy is engaged at first but then retrieves his bowl and sits back down with it. The PT hides a load of metal boxes in the stool, the boy is not very interested but eventually comes over. The PT positions him in half kneeling, hand-leads him to support himself with one hand, and hand-leads him to reach with a magnetic stick with the other and fetch one of the boxes. The boy looks at it briefly and then throws both the box and the stick out of his hands. The PT retrieves the stick and places it

back in his hands and tries to hand-lead him towards the stool again, but the resists and tries to throw the stick again.

The PT tries to move on to new activities, but the boy continues crying. When the PT starts playing with jumping frogs he is interested and calms down and pushes a couple of frogs down from the stool before he walks away into the kitchen where his family members are. The PT follows along and now brings him to the basement staircase. She wants him to practice going up the stairs but the boy cries in objection and sobs as he crawls up. She tries different ideas to engage him in play while moving in the stairs, much of the time hand-led by the PT. He is briefly engaged in these activities but quickly starts to object and eventually cries constantly as she guides his walking up the stairs the last time.

During the interview, the PT explains that she tries not to attend too much to his objections and that it is important to complete the activities with him. He usually cooperates better with her than he did this day. She also explains that her handling is aimed at making him work more with rotations and in the sagittal plane, because he is 'frontal' in his movement patterns.

Thoughts on therapeutic handling and merging with play

Although this session in packed with what could be fun play activities, they are very adult-driven and with a fixed plan of what the motor performance should be. While the boy shows play engagement whenever new activities are introduced, the PT's governing of things seems to quickly frustrate him. The PT's hand-leading/ guiding of movements also seem to override the opportunities for self-initiated movement and problem-solving. Although there are sequences in which the PT observes him moving on his own, the PT's handling is taking the lead of the movement rather than supporting and expanding the boy's self-initiated movements. The boy's objections increase throughout the session, one might wonder if a sense of his body being governed by the PT is a factor in this. Noteworthy, he does seem to disconnect to play/ increase his complaints whenever the PT is more hands-on with him.

P12-C24

Boy, 1y2m. Session in family home, mom and sibling at home but not involved in the session. The PT places the boy on a therapy ball in prone, and they do a game where the boy grabs a puzzle piece and then rolls forward on the ball to put it on the floor. The boy is engaged in the activity which is mainly hand-led by the PT. She then sits him up on the ball, bounces him a bit, and then changes the activity to putting the puzzle pieces together. She guides and supports the boy's shifts on the ball between prone to fetch pieces and sitting to puzzle with his left hand on a chair next to him. The PT hand-lead him in putting the pieces together. The boy is engaged in the activity. When the boy puts one of the pieces on his head, the PT picks up on this and makes it a play activity for a short while, before they return to puzzling it in place.

Next, she sits him up on a low stool and has him put the puzzle pieces away in a bag. She uses both her arms and legs to support him as he raises to stand and sits down on the stool as part of the activity. They now switch to play with beaded strings, the boy is very eager with putting them both around his own and the PT's neck, and the PT supports him in standing by use of her hand or between her own legs, with short sitting breaks on the stool. The bead activity seems to be very motivating for the boy and the PT embeds prolonged standing and stepping-onto-stool training into his fetching, hanging on and removal of beads.

Next the PT moves him to standing by a chair and invites him to continue cruising to the next chair. When he doesn't show signs of moving, she hand-leads him a little, upon which he continues to move on his own initiative. He turns around to a cabinet with doors and draws that they start opening and closing to hide shapes inside of it. The PT handles the boy in and out of squatting, places his hands against surfaces for support, reaches with his arms and rotates his trunk. By this, there is a mix of him moving by himself, her moving his body for him and them moving together. She maintains a dialogue with the boy and picks up on his signs of interest. But other times she seems to be initiating activities at her own will, most of the time is successfully engaging him in putting, stacking, or dumping shapes on the floor.

As he seems to be losing interest in the shapes, she asks if he is all done and makes him put them away in the container while cruising between the two chairs. After a couple of rounds the boy disengages and sits down. She handles him and guides him back up into standing, and make him put one more in the container, cheering him on as he completes the task. She then introduces a magic wand to play with, gives it to him and guides him in walking and the places him in standing with support from the coach behind him and herself sitting down in front of him to play with the wand. She handles him to help him balance and stand independently. The final activity is placing of beads in a small container, set up so he must squat, stand and rotate to both sides to place them. The boy is very engaged in this activity, and it continues until the session ends.

During the interview the PT tells that this boy is showing more initiative the last few monts and is now easily engaged in play activities with little objections to her handling of him. She was pleased to be able to work so much in standing and cruising activities, admitting that he needed a lot of support from her to continue working.

Thoughts on therapeutic handling and merging with play

This session is full of engaging play activities and tasks, but they are mostly initiated and led by the PT and there is not much time for the boy to explore things on his own and discover by himself what he wants to do. Also, the PT is eager with her use of hands and seems to have little hesitations with 'taking over' his movements and guiding him to move the way she wants him to. However, there are instances when this makes him reorganize his body/ take steps to position himself and better complete the task that he is doing. Although the boy plays along with all the activities introduced by the PT, the activity with the beads seems to be the most engaging one for him. Here he shows perseverance, wants to do many repetitions and also gets his dose of social feedback from the PT as part of the play activity. His eagerness to play also makes the PT's handling less 'intrusive', making him the 'leader of his movements' for most of the time and at the same time challenging his own movement repertoire in terms of squatting/standing/ reaching and using his arms while balancing.

P14-C28

Boy, 8m (CA 5m). Session in family home with granddad present but not very involved. They start out with the boy lying in supine on the floor. The PT holds a rattle up in his visual space, moves it slightly to the sides to see if he will follow it with his gaze, and also taps at his hands and feet to see if he will grab at it and 'get his feet going'. He grabs it with his right hand and handles it for a short while with both hands before the PT pulls it away from him and holds it up again for him to reach it. He is most active with his right hand, the PT reminds him about his left hand but ends up allowing him to grab it with his right.

Next she assists him in rolling over his right side into prone. He rolls himself back into supine, and granddad confirms that he will only roll prone to supine, not supine to prone on his own. After some reorganizing of blankets on the floor, the PT places the rattle on his right side, and pulls at his left leg and arm to assist him in rolling over. He rolls over and grabs the rattle. The PT pulls it out of his hands and holds it up for him to rotate his neck and follow it with his gaze. She then puts in down on the ground on his right side. The boy reaches and grabs a second toy, holds and mouths at it for a short while. The PT grabs it from him and moves it slightly out of his reach to the left. The boy loses interest, struggles a bit in prone before he rolls himself back into supine. The PT then asks 'do you want to do some sit-ups' with a cheerful voice, pulls him up and down a couple of times and then ends up with him in sitting. At this point the boy makes eye contact with granddad and smiles and babbles with him. The PT comments that it's nice to see that he's using his vision. The interaction continues, the boy is a bit wobbly, and the PT is there with her hand to correct him as he is about to lose his balance. Eventually he falls backwards into the PT's hand, and she leads the fall gently to the ground.

Next, she places him in prone and pulls out some strings of bead. He reaches out for them; she pulls them away and holds them up from the ground for him to reach. He grabs it with his right hand and rolls over to supine, then is helped back into prone by the PT but rolls back to supine again, all the while the PT holds the beads up for him in different positions. She practices rolling with him a couple of times, but after a while he loses interest with the beads. The PT continues to help him roll between supine and prone, eventually ending up in supine and playing peek-a-boo with a piece of cloth over his head. Next the PT pulls him back up to practice his sitting balance again, then moves him back into supine and again helps him roll into prone over his right side. He rolls back to supine, but the PT quickly helps him back to prone. Again, the boy rolls back to supine, the PT tries to roll him over again, he pushes back but then completes the roll, but rolls back to supine shortly thereafter. Assisted by the PT, the boy keeps going back and forth between supine and prone a couple of times more, before the PT sits him back up and the session is over.

During the interview the PT is talking about his development and skill level, choices of toys and activities and how she works as a therapist to get a good idea about the family's everyday life. There is little talk about how she reasons about her handling approach and the outcomes of it.

Thoughts on therapeutic handling and merging with play

In this session the boy is gradually becoming more interested and engaged in playing, he seems to go along with everything that is being done, occasionally engages in social interaction play but is also very content with his ongoing object play. The PT follows a track of 'double attention' throughout the session, checking out and stimulating the boy's motor skills on the one hand, and keeping up the conversation with granddad on the other. Along this 'double track' there are some salient moments when she focuses on the interaction with the boy, as a response she gets more babbling from him, and a more engaged facial expressions with good eye contact and smiles. But overall, she is mostly doing things to the boy – introducing, placing and removing toys/ shifting positions for him – rather than them interactively doing things together.

P15-C30

Girl, 11m. Session at PT office with mom present. The girl takes a bit of time to warm up, but after a while the PT makes contact with the girl, 'I heard you said ball', she says, and they fetch out a ball from the toy box. The girl is curious but remains in her sitting position. The PT guides her to support one hand on the floor and move out of sitting, the girl successfully transitions into prone and can now

push and crawls reciprocally after the ball. Next the PT picks the girl up and positions her in kneeling over her own thigh. The girl resist the position and the PT places her back on the floor: 'let's not make you angry this early in the session, let's wait a bit'.

Next the girl heads brought to knee-standing by a squared pillow. From this position the girl is able to rise to stand by herself. She continues to engage with ball play, in a 'dangling' standing position supported by the PT: 'you feel pretty sure that I'm holding you here, do you?' At one point the girl falls back into the PT's lap and is brought down on the floor, but she wants to get back up and is guided by the PT in her problem-solving of rising to stand. The girl continues to play and drops a toy to the floor. The PT guides her down towards kneeling to pick it up, but the girl sits herself all the way down on her bottom. From this position it is difficult for her to get back up, so the PT guides her to a mermaid-sitting and helps her up into kneeling and standing. The rest of the session is an examination of her hips and feet, with feedback and advice to mom on how to continue their follow up in everyday life.

During the interview the PT explains that this was more of a check-up session than an ongoing intervention, but that the use of play is just as relevant here and enables her to make her observations and assess the child's skill level.

Thoughts on therapeutic handling and merging with play

This session primarily has an examination focus, and play is used to create good situations for the PT to do her tasks. The girl is easily engaged, the PT is attentive to the child's signals and backs out of situations that seem to make the girl less comfortable. But still she is willing to push these boundaries in short sequences, but then makes an effort to re-establish trust and re-engage in mutual play afterwards. In the few situations when the PT tries to facilitate problem solving/ motor learning, play is a prime driver of the activity and the PT's guiding actions helps the girl achieve her goals, although not always in the manner that the PT planned for. But the PT is flexible in her approach and welcomes the girl's alternative movement choices.
